# Effects of β-mannanase supplementation on intestinal health and growth of nursery pigs

**DOI:** 10.1093/jas/skae052

**Published:** 2024-02-29

**Authors:** Ki Beom Jang, Young Ihn Kim, Marcos Elias Duarte, Sung Woo Kim

**Affiliations:** Department of Animal Science, North Carolina State University, Raleigh, NC 27695, USA; Department of Animal Science, North Carolina State University, Raleigh, NC 27695, USA; Department of Animal Science, North Carolina State University, Raleigh, NC 27695, USA; Department of Animal Science, North Carolina State University, Raleigh, NC 27695, USA

**Keywords:** inflammation, jejunum, β-mannanase, nursery pigs, oxidative stress, viscosity

## Abstract

Two experiments were conducted using 120 pigs to test the hypothesis that supplementation of β-mannanase could reduce digesta viscosity, enhance nutrient digestion, and improve intestinal health and growth of nursery pigs. In experiment 1, 48 crossbred barrows were randomly allotted to four treatments with increasing levels of β-mannanase at 0, 200, 400, and 600 U/kg in feeds. All pigs were euthanized on day 12 to collect jejunal digesta to measure digesta viscosity and ileal digesta to measure apparent ileal digestibility (AID) of dry matter (DM), gross energy (GE), neutral detergent fiber (NDF), and acid detergent fiber (ADF). In experiment 2, 72 nursery pigs were randomly allotted to three treatments with increasing levels of β-mannanase at 0, 400, and 600 U/kg in feeds. Plasma collected on day 9 was used to measure tumor necrosis factor-α (TNF-α), immunoglobulin G (IgG), malondialdehyde (MDA), and protein carbonyl (PC). All pigs were euthanized on day 10 to collect duodenal and jejunal tissues to evaluate the production of TNF-α, IL-6, and MDA, morphology, crypt cell proliferation, and expression of tight junction proteins in the jejunum. Data were analyzed using the MIXED procedure for polynomial contrasts and the NLMIXED procedure for broken-line analysis of SAS. In experiment 1, β-mannanase supplementation tended to have quadratic effects on digesta viscosity (*P* = 0.085) and AID of GE (*P* = 0.093) in the pigs. In experiment 2, jejunal digesta viscosity of the pigs was reduced (*P* < 0.05) when β-mannanase was supplemented at 360 U/kg of feed. β-Mannanase supplementation linearly reduced (*P* < 0.05) TNF-α, IgG, MDA, and PC in the duodenum, and TNF-α, IgG, and MDA in the jejunum of the pigs. β-Mannanase supplementation linearly increased (*P* < 0.05) villus height to crypt depth ratio and crypt cell proliferation in the jejunum. β-Mannanase supplementation tended to linearly improve (*P* = 0.083) expression of zonula occludens-1 in the jejunum. In conclusion, supplementation of β-mannanase at 360 U/kg reduced the digesta viscosity and up to 600 U/kg positively affected intestinal health and growth of pigs by reducing inflammation and oxidative stress whilst enhancing structure and barrier function in the jejunum.

## Teaser

β-Mannanase effectively reduced digesta viscosity through hydrolysis of β-mannan in feeds and thus enhanced intestinal health of nursery pigs by reducing inflammation and oxidative stress whilst improving intestinal barrier function in the jejunum.

## Introduction

Nursery pigs are commonly susceptible to the challenges that can negatively affect their immature digestive systems and potentially impair growth and health (Kim and Duarte, 2021). The small intestine is a crucial part of the gastrointestinal tract responsible for feed digestion, absorption, and protection from toxins and external invasions (Lallès et al., 2007; Zheng et al., 2021). One of the significant limitations for newly weaned pigs is their inability to digest complex carbohydrates, including non-starch polysaccharides (NSP) found in feeds. β-Mannans and β-galactomannans are NSP abundant in protein supplements such as copra meal, palm kernel meal, and soybean meal (Kiarie et al., 2013; Jang et al., 2023a). Once ingested, β-mannans exhibit hygroscopic properties, increasing digesta viscosity in the small intestine, which can hinder the movement of digesta through the intestine, impede the access of digestive enzymes to digesta, reduce the surface area available for nutrient digestion, and impair nutrient absorption and growth of pigs (Jha and Mishra, 2021; Kiarie et al., 2021).

β-Mannanase specifically targets β-mannans and β-galactomannans, hydrolyzing β-1,4-mannosidic bonds (Ximenes et al., 2005; Tiwari et al., 2018) resulting in reduced digesta viscosity and improved nutrient digestibility (Jeon et al., 2019; Genova et al., 2023). Furthermore, the hydrolysis of β-mannans into mannan oligosaccharides can provide prebiotic effects (Halas and Nochta, 2012; Agazzi et al., 2020) and inactivate toxic compounds such as aflatoxin (Kim et al., 2016) benefiting the intestinal environment in nursery pigs. Beneficial effects of β-mannanase supplementation have been shown to be related to reduced digesta viscosity, enhanced NSP digestion, and improved growth performance (Balasubramanian et al., 2018), whereas its potential mechanisms related to mucosal health warrant investigation. Therefore, it was hypothesized that supplementation of β-mannanase could reduce digesta viscosity and increase nutrient digestion by enhancing intestinal health thereby improving the growth performance of nursery pigs. To test the hypothesis, the objective of this study was to evaluate the effects of increasing supplementation levels of β-mannanase on inflammatory status, oxidative stress status, humoral immune status, structural damages and repairing, and barrier function in the jejunum as well as nutrient digestibility and growth performance of nursery pigs.

## Materials and Methods

The protocol of this experiment was reviewed and approved by the Institutional Animal Care and Use Committee at North Carolina State University (Raleigh, NC, USA). Two experiments were conducted under the guidelines in the care and management of animals (Federation of Animal Science Societies, 2020) at research facilities at North Carolina State University (Raleigh, NC, USA).

### Animals and experimental feeds

In experiment 1, 40 barrows were weaned at 21 d of age and fed a common diet for 21 d prior to the initiation of the study. After 21 d feeding of a common feed, barrows with 15.7 ± 1.1 kg of body weight (BW) were allotted to four treatments based on a randomized complete block design with initial BW (heavy and light) as a block. Each treatment had 12 replicates and the pigs were individually placed in metabolism pens (0.6 m × 1.8 m) equipped with a stainless-steel feeder attached to the front of the cage, nipple water next to the feeder, and slatted flooring (Passos et al., 2015a). β-Mannanase was supplemented at 0, 250, 500, and 750 mg/kg into the basal diet to provide 0, 200, 400, and 600 U/kg, respectively. The basal feeds contained 58.2% corn, 27.0% soybean meal, 10.0% corn distiller’s dried grains with solubles (DDGS), and 4.5% others (Table 1). Pigs were provided with the experimental feeds at a fixed amount based on the BW of pigs (daily feed allowance = 0.09 × BW^0.75^ kg) for 12 d. Feeds were provided two times per day (0700 and 1700 hours). The BW of individual pigs and daily feed disappearance were recorded to determine average daily gain (ADG), average daily feed intake (ADFI), and feed efficiency (gain to feed ratio [G:F]) until day 11 and all pigs were euthanized on day 12.

**Table 1.** Composition of experimental feeds^1^ used in experiment 1 (as-fed basis)

Item	Basal feed
Feedstuff, %	
Corn, yellow	58.22
Soybean meal, 48% crude protein	27.00
Corn DDGS^2^	10.00
l-Lys HCl	0.20
Salt	0.30
Vitamin and mineral premix^3^	0.18
Monocalcium phosphate	0.50
Limestone	1.30
Poultry fat	2.00
Titanium dioxide	0.30
Total	100.00
Calculated composition	
ME, Mcal/kg	3.34
Crude protein, %	20.63
SID^4^ Lys, %	1.02
SID Thr, %	0.62
SID Trp, %	0.21
Total mannan^5^, %	0.31
Analyzed composition	
Dry matter, %	90.42
Crude protein, %	21.41
Ca, %	0.55
P, %	0.63
NDF^6^, %	12.71
ADF^7^, %	3.54

^1^β-Mannanase was added replacing the corn at 0, 250, 500, or 750 mg/kg of the basal feed to adjust as 0, 200, 400, or 600 U/kg. Analyzed β-mannanase activities in the experimental diet were 0, 265 ± 3, 438 ± 3, or 623 ± 5 U/kg of feeds.

^2^DDGS: distiller’s dried grains with solubles.

^3^The vitamin premix provided per kg of complete diet: 8,433 IU of vitamin A, 1,202 IU of vitamin D_3_ as activated animal sterol, 48 IU of vitamin E, 4.0 mg of vitamin K as menadione dimethylpyrimidinol bisulfate, 6.0 mg of riboflavin, 36.2 mg of niacin, 24.1 mg of d-pantothenic acid as calcium pantothenate, 1.8 mg of folic acid, 0.24 mg of d-biotin, 0.031 mg of vitamin B_12_. The trace mineral premix provided per kg of complete diet: 16.5 mg of Cu as CuSO_4_, 0.3 mg I as ethylenediamine dihydriodide, 165 mg of Fe as FeSO_4_, 40 mg of Mn as MnSO_4_, 0.3 mg of Se as Na_2_SeO_3_, and 165 mg of Zn as ZnO.

^4^SID: standardized ileal digestible.

^5^Calculated based on Kiarie et al. (2021).

^6^NDF: neutral detergent fiber.

^7^ADF: acid detergent fiber.

In experiment 2, 72 pigs were weaned at 21 d of age and fed a common diet for 24 d prior to the initiation of the study. The common diet contained the nutrients meeting the requirement for the pigs suggested by NRC (2012). After 24 d feeding of a common diet, pigs (36 barrows and 36 gilts; initial BW 15.5 ± 2.3 kg) were allotted to three treatments based on a randomized complete block design with initial BW (heavy, middle, and light) and sex (barrows and gilts) as blocks. Each treatment had 24 replicates and the pigs were individually placed in pens (1.4 m × 3.9 m) equipped with a stainless-steel feeder attached to the front of the cage, nipple water next to the feeder, and slatted flooring (Choi et al., 2023). β-Mannanase was supplemented at 0, 500, and 750 mg/kg into the basal diet to provide 0, 400, and 600 U/kg, respectively. The basal feeds contained 51.9% corn, 33.0% soybean meal, 10.0% corn DDGS, and 5.1% others (Table 2). Pigs were provided with the experimental feeds and water ad libitum for 10 d. The BW of individual pigs and feed disappearance were recorded to determine ADG, ADFI, and G:F. All pigs were euthanized on day 10 immediately after recording the BW and feed disappearance.

**Table 2.** Composition of experimental feeds^1^ used in experiment 2 (as-fed basis)

Item	Basal feed
Feedstuff, %	
Corn, yellow	51.87
Soybean meal, 48% crude protein	33.00
Corn DDGS^2^	10.00
l-Lys HCl	0.30
dl-Met	0.05
l-Thr	0.05
Salt	0.30
Vitamin and mineral premix^3^	0.18
Dicalcium phosphate	0.55
Limestone	1.20
Poultry fat	2.00
Total	100.00
Calculated composition	
ME, Mcal/kg	3.39
Crude protein, %	23.16
SID^4^ Lys, %	1.25
SID Thr, %	0.75
SID Trp, %	0.24
Total mannan^5^, %	0.62
Analyzed composition	
Dry matter, %	91.28
Crude protein, %	21.41
Ca, %	0.65
P, %	0.60
NDF^6^, %	8.60
ADF^7^, %	4.01

^1^β-Mannanase was added at 0, 500, or 750 mg/kg of basal feed to adjust as 0, 400, or 600 unit/kg. Analyzed β-mannanase activities in the experimental diet were 0, 477 ± 32, or 697 ± 21 U/kg of feeds.

^2^DDGS, distiller’s dried grains with solubles.

^3^The vitamin premix provided per kg of complete diet: 8,433 IU of vitamin A, 1,202 IU of vitamin D_3_ as activated animal sterol, 48 IU of vitamin E, 4.0 mg of vitamin K as menadione dimethylpyrimidinol bisulfate, 6.0 mg of riboflavin, 36.2 mg of niacin, 24.1 mg of d-pantothenic acid as calcium pantothenate, 1.8 mg of folic acid, 0.24 mg of d-biotin, 0.031 mg of vitamin B_12_. The trace mineral premix provided per kg of complete diet: 16.5 mg of Cu as CuSO_4_, 0.3 mg I as ethylenediamine dihydriodide, 165 mg of Fe as FeSO_4_, 40 mg of Mn as MnSO_4_, 0.3 mg of Se as Na_2_SeO_3_, and 165 mg of Zn as ZnO.

^4^SID, standardized ileal digestible.

^5^Calculated based on Kiarie et al. (2021).

^6^NDF, neutral detergent fiber.

^7^ADF, acid detergent fiber.

The experimental feeds used in experiments 1 and 2 were formulated to provide nutrients to meet or exceed requirements suggested by NRC (2012) and formulated at the North Carolina Feed Educational Unit (Raleigh, NC, USA). All diets were in mash form. The supplemental levels of β-mannanase were set up based on the significant biological responses in previous studies (Yoon et al. 2010; Lv et al., 2013; Kim et al., 2017). Beta-mannanase (800,000 U/kg; CTCBIO Inc., Seoul, South Korea) was produced through the fermentation by *Bacillus subtilis* isolate WL-7 (GenBank AAT27435.1) on Luria broth. One unit of β-mannanase activity was defined as the amount of enzyme required to release 1 µmole of mannose reducing sugars equivalents per minute from 1.0% locust bean gum in 200 mM sodium phosphate buffer, pH 6.0 at 50 °C.

### Sample collection

In experiment 1, at the end of the studies, all pigs were euthanized by exsanguination after the penetration of a captive bolt to the head to collect digesta samples. Immediately after the euthanasia, the ileal portion (a portion of 20 cm prior to the ileocecal connection) of the small intestine was used to obtain digesta in the ileum. The distal portion of the jejunum (20 cm) was also used to collect jejunal digesta to measure viscosity using a viscometer (Brookfield Digital Viscometer, Model DV-II Version 2.0, Brookfield Engineering Laboratories, Inc., Stoughton, MA). Digesta from the ileum were stored in a sterile container and kept frozen at −20 °C.

In experiment 2, blood samples were collected (10 mL) using vacutainer tubes containing dipotassium EDTA (BD, Franklin Lakes, NJ) from the jugular vein of pigs in all pens on day 9. Blood sampling was done without prior fasting at 0900 hours when pigs were active in eating behavior (Hyun et al., 1997; Nyachoti et al., 2004). The tubes were centrifuged at 3,000 × *g* for 15 min and then the plasma was aliquoted into 1.5 mL tubes and stored at −80 °C until analysis. On the last day of the experiment, all pigs were euthanized by the penetration of a captive bolt followed by exsanguination. After euthanasia, tissues from the duodenum (5 cm after the pyloric-duodenal junction) and distal jejunum (3 m distal to the pyloric-duodenal junction) were collected and flushed with sterile saline solution. The first 10 cm was used to collect jejunal mucosa by scraping the mucosal layer in the jejunum using a glass microscope slide and the remaining 5 cm was stored in 10% formalin buffer for histological evaluation. Duodenal and jejunal mucosa and plasma samples were used to measure tumor necrosis factor-α (TNF-α) and immunoglobulin G (IgG) as indicators of immune status and malondialdehyde (MDA), and protein carbonyl (PC) as indicators of oxidative stress status.

### Viscosity of jejunal digesta

Viscosity was immediately measured in the same day after obtaining jejunal digesta based on the methods described by Passos et al. (2015b). The jejunal digesta were homogenized and then transferred to 15 mL tubes (each sample repeated four times). The tubes were centrifuged at 1,500 × *g* for 5 min at 4 °C and then the supernatant was again centrifuged at 10,000 × *g* for 10 min at 4 °C. The second supernatant was placed in ice to measure viscosity immediately. A viscometer (Brookfield Digital Viscometer, Model DV-II Version 2.0, Brookfield Engineering Laboratories, Inc.) was set at 25 °C, and 1.0 mL of the supernatant was placed in the viscometer.

### Apparent ileal digestibility

Titanium dioxide (TiO_2_) was added to the feeds at 0.3% as an indigestible external marker for the last 5 days of the first experiment. Titanium dioxide (TiO_2_) concentrations of the diets and ileal digesta were determined by the procedure described in Chen et al. (2017). The apparent ileal digestibility (AID) of nutrients was calculated according to the following equation:


$$\begin{array}{l} AID\;of\;nutrients,\;\%=\left[1-\left(\frac{TiO_{2diet}}{TiO_{2digesta}}\right)\times \left(\frac{NUTR_{digesta}}{NUTR_{diet}}\right)\right]\times 100 \end{array}$$

where TiO_2diet_ and TiO_2digesta_ are the TiO_2_ contents in the diet and ileal digesta, respectively (g/kg; dry matter [DM] basis); and NUTR_digesta_ and NUTR_diet_ are the nutrient contents in the ileal digesta and diet, respectively (DM basis). Ileal digesta were stored at −20 °C and freeze-dried (24D 48, Virtis, Gardiner, NY). The feeds and freeze-dried digesta samples were ground and analyzed for TiO_2_ concentration following the methodology described by Williams et al. (1962), neutral detergent fiber (NDF) following Van Soest et al. (1991), and acid detergent fiber (ADF) based on (Method 973.18, AOAC, 2006). AID of NDF and ADF were calculated using titanium concentration in the feeds and digesta.

### Intestinal morphology and Ki-67 in crypt cells

Two parts of mid-jejunum were fixed in 10% buffered formalin for 48 h after tissue sampling, and then transferred to a 70% ethanol solution and sent to North Carolina State University Histology Laboratory (College of Veterinary Medicine, Raleigh, NC) for dehydration, embedment, and hematoxylin and eosin staining as well as immunohistochemistry for staining Ki67^+^ antibody to measure the proliferation of enterocytes according to Deng et al. (2022). Villus height and crypt depth (VH:CD) were measured using a camera (Infinity 2-2 digital CCD) attached to a microscope (Olympus CX31, Lumenera Corporation, Ottawa, Canada) as described in previous studies (Deng et al., 2022; Moita and Kim, 2023). Lengths of 15 well-oriented intact villi and their associated crypts were measured in each slide. The villi length was measured from the top of the villi to the villi-crypt junction, and the CD was measured from the villi-crypt junction to the bottom of the crypt. Then, the VH:CD ratio was calculated. Images of 15 intact crypts from each slide were cropped and the Image JS software was used for calculating the ratio of Ki-67 positive cells to total cells in the crypt (%) as described in previous studies (Duarte et al., 2019; Kim et al., 2019). Morphological evaluation and crypt cell proliferation were executed by the same person.

### Inflammatory cytokine, immunoglobulin, and oxidative damage products

Duodenal and jejunal mucosa samples (1 g) were taken and added with 2 mL of phosphate-buffered saline solution into 5 mL polypropylene tubes. The mucosa samples were homogenized using a tissue homogenizer (Tissuemiser, Thermo Fisher Scientific Inc., Rockford, IL) for 30 s on ice and transferred to a new 2 mL microcentrifuge tube for centrifugation for 15 min at 14,000 × *g* at 4 °C as described in previous studies (Jang et al., 2020; Duarte et al., 2023). The supernatant was aliquot into four sets of 0.25 mL into polypropylene tubes and stored at −80 °C for further analysis. The concentration of total protein was analyzed by using Pierce BCA Protein Assay Kit (#23225, Thermo Fisher Scientific, Waltham, MA) following the manufacturer protocol. Mucosa samples were diluted (1:60) to reach the working range of 20 to 2,000 μg/mL. The absorbance was measured at 562 nm. The total protein concentration was calculated by the standard curve and used to normalize the concentrations of protein measures. Concentrations of TNF-α and IgG in the mucosa and plasma samples were determined using ELISA kits for TNF-α (#PTA00, R&D Systems, Minneapolis, MN) and IgG (#E101-104, Bethyl Laboratories Inc., Montgomery, TX) following the manufacturer’s protocols. The concentrations of MDA and PC were measured by commercial assay kits (#STA-330 and STA-310, respectively, Cell Biolabs, Inc., San Diego, CA) following the manufacture protocols. The working range of TNF-α standards was 0 to 1,500 pg/mL. The minimum detectable TNF-α was 2.8 pg/mL, and the intra- and interassay coefficients of variation (CV) were 4.1% and 8.3%, respectively. The working range of IgG standards was 0 to 500 ng/mL. The minimum detectable IgG was 0.69 ng/mL, and the intra- and interassay CV were 4.4% and 5.7%, respectively. The working range of MDA standards was 0 to 125 μM. The minimum detectable MDA was 0.98 μM and the intra-assay and interassay CV were 7.8% and 7.3%, respectively. The working range of PC standards was 0 to 7.5 nmol/mg. The minimum detectable PC was 0.38 nmol/mg and the intra-assay and interassay CV were 5.0% and 3.0%, respectively.

### Tight junction proteins

Four samples of jejunal tissue in each treatment were used to measure tight junction protein as described by Kim et al. (2019). Tissue samples (50 mg) from the jejunum were weighed and suspended into 0.5 mL RIPA lysis and extraction buffer containing 5 µL protease inhibitor cocktail. Tissue samples were homogenized (Tissuemiser; Thermo Fisher Scientific Inc) on ice. The homogenate was centrifuged at 10,000 × *g* at 4 °C for 10 min to collect supernatant. The protein concentration of the supernatant was adjusted to 2 µg/µL by using a BCA protein assay as mentioned above. The adjusted supernatant was denatured at 100 °C for 5 min in the water bath and was loaded in each well for SDS-PAGE. After SDS-PAGE, the gel was moved to a polyvinylidene difluoride membrane for transferring a target protein to the membrane. Protein was electrophoretically transferred at 90 mV for 1 h. These were then blocked in 5% skim milk, and incubated overnight at 4 °C with primary antibodies against claudin (Sigma, St. Louis, MO) diluted 1:400, occludin (Abcam, Cambridge, United Kingdom) diluted 1:100, zonula occludens-1 (ZO-1; Santa Cruz Biotechnology, Santa Cruz, CA) diluted 1:150, and β-actin (Cell Signaling Technology, Danvers, MA). The membrane was subsequently washed and incubated for 1 h at room temperature with horseradish-conjugated secondary antibodies. Immuno-positive bands were visualized by a chemiluminescent method (ECL, Amersham). The band density of tight junction proteins and housekeeping protein were measured with an image analyzer software (LI-COR Biosciences, Lincoln, NE). Each experiment sample was run in triplicate. Exposure times were adjusted so that the darkest bands did not saturate the film. The band densities were determined by summing all the gray level pixel values, after background subtraction, between the boundaries of the bands denoted by the lines. To avoid errors due to differences in signal intensity between the three blots, the average value of triplicate band density was used to calculate the relative band density of tight junction proteins. Relative band density was determined by dividing the band density of the tight junction protein by the band density of β-actin.

### Statistical analysis

A randomized complete block design was used in this study with blocking criteria (in experiment 1, initial BW; in experiment 2, sex and initial BW). In the model, a fixed effect was the β-mannanase supplementation and random effects were sex and initial BW. The data were analyzed using Mixed procedure of SAS (version 9.4, SAS Inst., Inc., Cary, NC). In both studies, a pig was the experimental unit. Polynomial contrasts were pre-planned to evaluate the dose–response to β-mannanase supplementation. The contrasts were determined using the Interactive Matrix Language (IML) procedure of SAS to generate coefficients for the unevenly spaced orthogonal contrasts. These coefficients generated by the IML procedure were then used in the Mixed procedure for contrasts. The viscosity data was further analyzed using the NLMIXED procedure to determine the breakpoint to obtain the optimal β-mannanase supplemental level, as previously described (Jang et al., 2021). The predictor was set by multiplying the β-mannanase inclusion (U/kg of feed) with the overall ADFI to account for the feed consumption of the animals through the experimental period (U/d). The *P* values less than 0.05 were considered statistically significant and between 0.05 and 0.10 were considered tendency.

## Results

### Experiment 1

The initial BW of pigs at the beginning of the experiment was 15.5 kg and there was no difference among treatments (Table 3). Increasing supplemental levels of β-mannanase from 0 to 600 U/kg in the feeds tended to linearly reduce (*P* = 0.076) digesta viscosity (5.23 to 4.38 centipoise [cP]) in the jejunum of pigs (Table 4). Increasing supplemental levels of β-mannanase from 0 to 600 U/kg in the feeds tended to have quadratic effects (*P* = 0.085) on digesta viscosity (minimum 3.87 cP at 419 U/kg of β-mannanase) in the jejunum of pigs. Increasing supplemental levels of β-mannanase from 0 to 600 U/kg in the feeds also tended to have quadratic effects (*P* = 0.093) on AID of gross energy (minimum 58.2% at 254 U/kg of β-mannanase) in the pigs. However, there was no difference in AID of DM, NDF, and ADF in nursery pigs by increasing supplemental levels of β-mannanase from 0 to 600 U/kg.

**Table 3.** Growth performance of nursery pigs in experiment 1

Item	β-mannanase, U^1^/kg				SEM	*P* value	
	0	200	400	600		Linear	Quadratic
Body weight, kg							
day 0	15.7	15.9	15.6	15.7	0.8	0.765	0.771
day 11	19.5	19.6	19.3	19.4	0.9	0.582	0.817
ADG, g/d	345	342	339	336	23	0.606	0.993
ADFI, g/d	736	748	716	732	31	0.263	0.833
G:F	0.47	0.46	0.48	0.46	0.03	0.828	0.809

^1^One unit of β-mannanase activity is defined as the amount of enzyme required to releases 1 μmol of mannose reducing sugars equivalents per minute from locust bean gum (1.0%) in sodium phosphate buffer (200 mM), pH 6.0 at 50°C.

**Table 4.** Jejunal digesta viscosity and AID in nursery pigs fed with β-mannanase in experiment 1^1^.

Item	β-mannanase, U^2^/kg				SEM	*P* value	
	0	200	400	600		Linear	Quadratic
Jejunal digesta viscosity, cP^3^	5.23	4.42	3.83	4.38	0.42	0.076	0.085
AID^4^, %							
Dry matter	66.0	55.7	66.3	65.1	3.7	0.571	0.134
Energy	64.3	53.4	64.5	64.7	3.9	0.395	0.093
NDF^5^	35.0	36.2	35.0	33.7	8.4	0.800	0.770
ADF^6^	20.3	23.6	20.8	19.0	8.9	0.785	0.630

^1^Each value in the table represents means of 12 pigs (1 pig per metabolic cage).

^2^One unit of β-mannanase activity was defined as the amount of enzyme required to releases 1 µmole of mannose reducing sugars equivalents per minute from 1.0% locust bean gum in 200 mM sodium phosphate buffer, pH 6.0 at 50 °C.

^3^Centipoise.

^4^AID: apparent ileal digestibility.

^5^NDF: neutral detergent fiber.

^6^ADF: acid detergent fiber.

### Experiment 2

**Figure 1.**
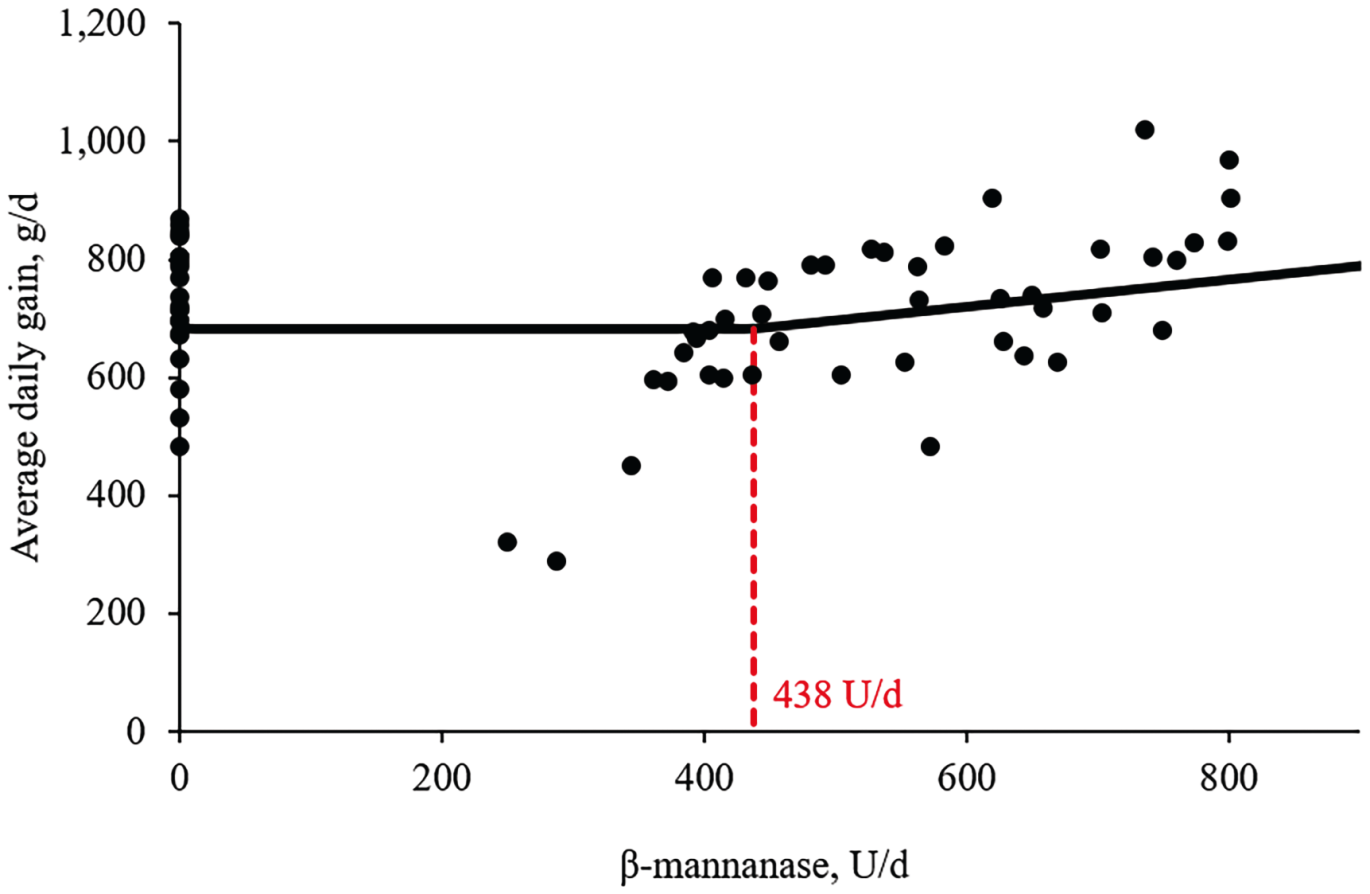
Changes in the ADG (g/d) with supplementation of β-mannanase using a broken-line analysis. The breakpoint was 438 U/d of β-mannanase supplementation when ADG was 683 g/d. The equation for ADG was Y = 683 − 0.41 × zl; if β-mannanase supplementation is lower than the breakpoint, then z = 0; if β-mannanase supplementation is higher than the breakpoint, then zl = breakpoint − β-mannanase supplementation. The *P* values for the plateau was <0.0001, for the slope was 0.0415, and for the breaking point was 0.0003. The breakpoint was converted from 438 U/d to 386 U/kg of feed by dividing it by the overall average feed intake (1.133 kg/d).

**Figure 2.**
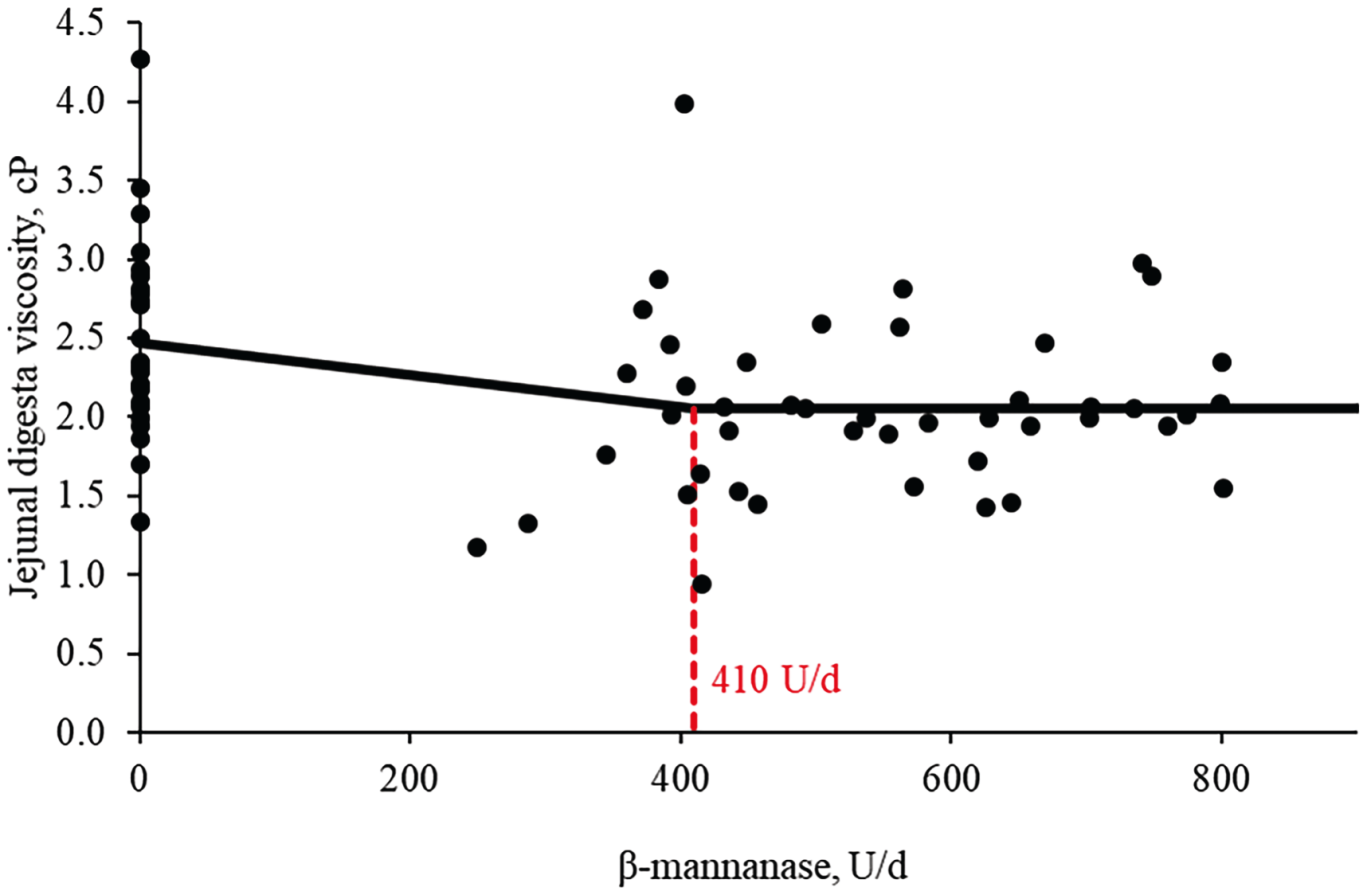
Changes in the jejunal digesta viscosity (centipoise [cP]) with supplementation of β-mannanase using a broken-line analysis. One cP is equal to the viscosity of water (=0.01 dyne*s/cm^2^). The breakpoint was 410 U/d of β-mannanase supplementation when the digesta viscosity was 2.06 cP. The equation for jejunal digesta viscosity was *Y* = 2.058 − 0.001 × zl; if β-mannanase supplementation is higher than the breakpoint, then *z* = 0; if β-mannanase supplementation is lower than the breakpoint, then *z*l = breakpoint − β-mannanase supplementation. The *P* value for the plateau was <0.001, for the slope was 0.021, and for the breaking point was 0.014. The breakpoint was converted from 410 U/d to 360 U/kg of feed by dividing it with the overall average feed intake (1.13 kg/d).

Increasing supplemental levels of β-mannanase from 0 to 600 U/kg in the feeds tended to have quadratic effects (*P* = 0.080) on ADG of nursery pigs (Table 5). However, there was no difference in BW on day 10, ADFI, and G:F in the pigs by increasing supplemental levels of β-mannanase from 0 to 600 U/kg in the feeds. According to broken-line analysis, ADG was increased (*P* < 0.05) when β-mannanase was supplemented over 386 U/kg of feeds (Figure 1). Increasing supplemental levels of β-mannanase from 0 to 600 U/kg in the feeds linearly reduced (*P* < 0.05) digesta viscosity (2.50 to 2.10 cP) in the jejunum of pigs. According to broken-line analysis, jejunal digesta viscosity of the pigs was reduced (*P* < 0.05) when β-mannanase was supplemented at 360 U/kg of feed (Figure 2).

**Table 5.** Growth performance and jejunal digesta viscosity of nursery pigs fed with β-mannanase in experiment 2.

Item	β-mannanase, U^1^/kg			SEM	*P* value	
	0	400	600		Linear	Quadratic
Body weight, kg						
day 0	15.4	15.5	15.5	0.8	0.546	0.424
day 10	22.8	22.5	23.0	1.0	0.574	0.192
ADG, g/d	737	699	750	28	0.632	0.080
ADFI, g/d	1,162	1,105	1,132	39	0.507	0.295
G:F	0.64	0.64	0.67	0.02	0.192	0.383
Jejunal digesta viscosity, cP^2^	2.50	2.09	2.10	0.15	0.022	0.170

^1^One unit of β-mannanase activity is defined as the amount of enzyme required to releases 1 μmol of mannose reducing sugars equivalents per minute from locust bean gum (1.0%) in sodium phosphate buffer (200 mM), pH 6.0 at 50 °C.

^2^cP: centipoise (1 cP = 1/100 dyne s/cm^2^).

Increasing supplemental levels of β-mannanase from 0 to 600 U/kg in the feeds linearly reduced (*P* < 0.05) TNF-α (5.81 to 3.81 ng/g of protein), IgG (1.44 to 1.07 mg/g), and MDA (1.46 to 1.26 μmol/g) in the duodenum of pigs (Table 6). Increasing supplemental levels of β-mannanase from 0 to 600 U/kg in the feeds also tended to have quadratic effects on IgG (*P* = 0.081) and PC (*P* = 0.093) in the duodenum of pigs. Increasing supplemental levels of β-mannanase from 0 to 600 U/kg in the feeds linearly reduced (*P* < 0.05) TNF-α (6.23 to 4.19 ng/g of protein), IgG (1.20 to 0.80 mg/g), MDA (1.06 to 0.69 μmol/g), and PC (7.11 to 4.22 μmol/g) in the jejunum of pigs. Increasing supplemental levels of β-mannanase from 0 to 600 U/kg in the feeds linearly reduced (*P* < 0.05) TNF-α (167.4 to 81.0 pg/mL) and IgG (1.54 to 1.03 pg/mL) in plasma of the pigs. Increasing supplemental levels of β-mannanase from 0 to 600 U/kg in the feeds had quadratic effects (*P* < 0.05) on MDA and tended to have quadratic effects (*P* = 0.071) on TNF-α in the plasma of pigs.

**Table 6.** Immune response and oxidative stress status in the duodenum, jejunum, and plasma of nursery pigs fed with β-mannanase in experiment 2.

Item^1^	β-mannanase, U^2^/kg			SEM	*P* value	
	0	400	600		Linear	Quadratic
Duodenum						
TNF-α, pg/mg protein	5.81	5.19	3.81	0.58	0.033	0.342
IgG, μg/mg protein	1.44	1.45	1.07	0.20	0.038	0.081
MDA, nmol/mg protein	1.46	1.31	1.26	0.10	0.046	0.859
PC, nmol/mg protein	3.71	3.89	3.22	0.30	0.249	0.093
Jejunum						
TNF-α, pg/mg protein	6.23	5.51	4.19	0.79	0.046	0.438
IgG, μg/mg protein	1.20	1.07	0.80	0.10	0.008	0.250
MDA, nmol/mg protein	1.06	0.96	0.69	0.12	0.002	0.123
PC, nmol/mg protein	7.11	4.61	4.22	0.41	<0.001	0.268
Plasma						
TNF-α, pg/mL	167.4	69.9	81.0	19.8	<0.001	0.071
IgG, mg/mL	1.54	1.35	1.03	0.12	0.004	0.275
MDA, nmol/mL	19.8	11.9	17.6	2.2	0.187	0.011
PC, nmol/mg protein	3.76	3.33	3.68	0.44	0.737	0.375

^1^TNF-α: tumor necrosis factor-α; IgG: immunoglobulin G; MDA: malondialdehyde; PC: protein carbonyl.

^2^One unit of β-mannanase activity is defined as the amount of enzyme required to releases 1 μmol of mannose reducing sugars equivalents per minute from locust bean gum (1.0%) in sodium phosphate buffer (200 mM), pH 6.0 at 50°C.

**Figure 3.**
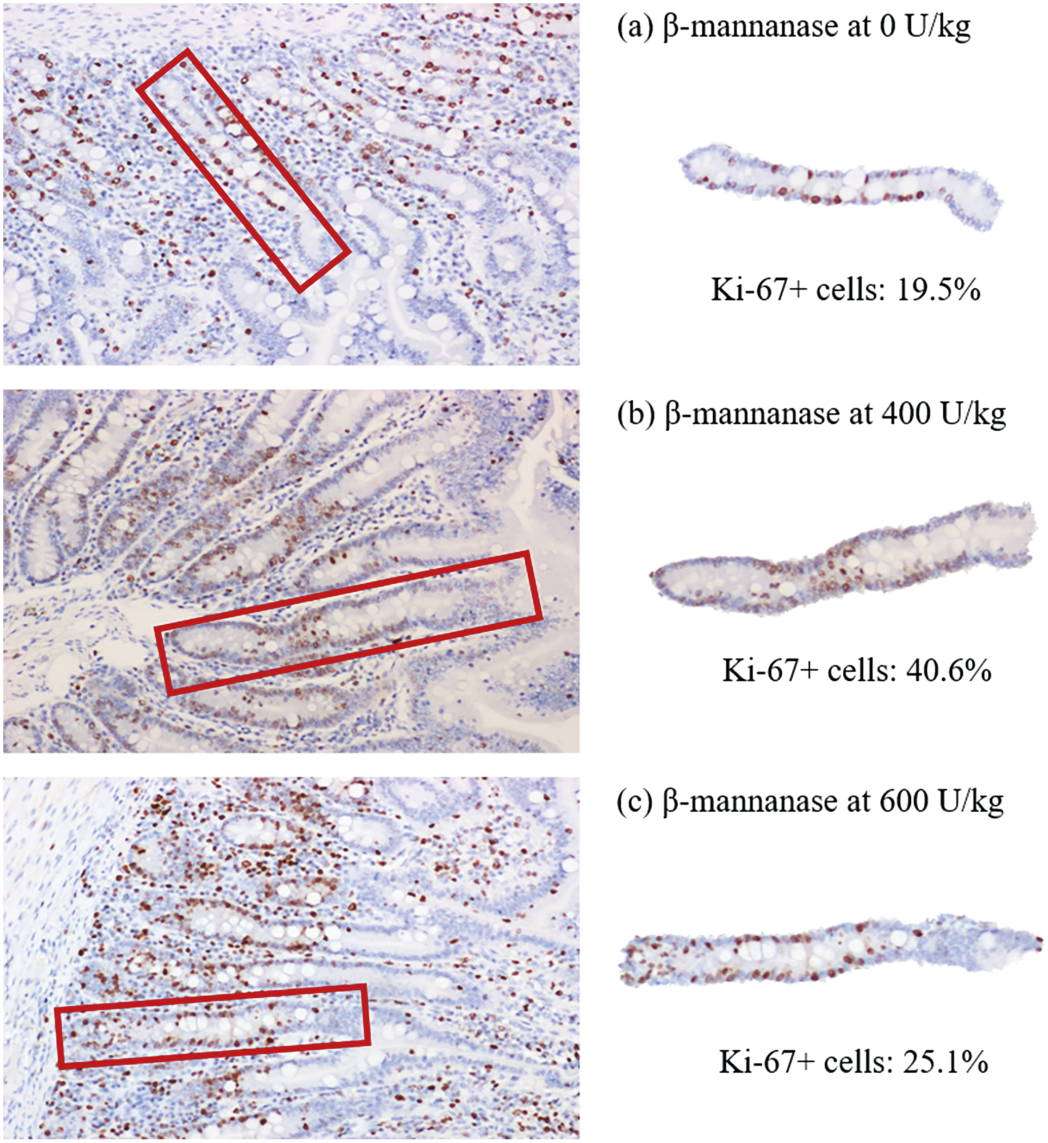
Representing images of crypts and crypt cells expressing Ki-67 proteins in the jejunum of pigs fed diets with (a) 0 U/kg of β-mannanase, (b) 400 U/kg, and (c) 600 U/kg. Using immunohistochemistry, cells expressing Ki-67 protein are shown in dark round dots. Percent of proliferating cells in a crypt is measured.

**Figure 4.**
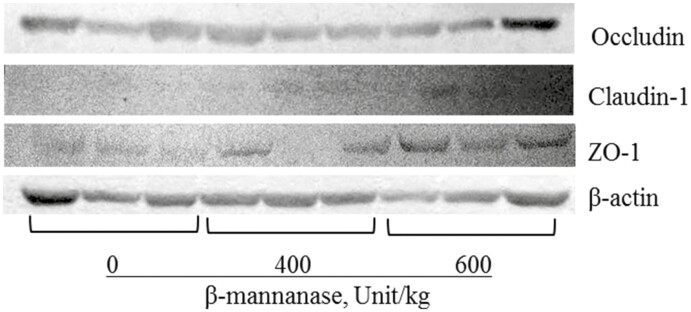
Tight junction proteins in the jejunum of nursery pigs fed with β-mannanase in experiment 2.

Increasing supplemental levels of β-mannanase from 0 to 600 U/kg in the feeds linearly increased (*P* < 0.05) VH (579 to 651 μm) and CD (281 to 301 μm) in the duodenum of nursery pigs (Table 7). Increasing supplemental levels of β-mannanase from 0 to 600 U/kg in the feeds linearly increased (*P* < 0.05) VH (426 to 516 μm), CD (175 to 246 μm), VH:CD (2.48 to 2.13) in the jejunum of pigs. Increasing supplemental levels of β-mannanase from 0 to 600 U/kg in the feeds linearly increased (*P* < 0.05) crypt cell proliferation (29.3 to 35.5%) and also had quadratic effects (*P* < 0.05) on crypt cell proliferation in the jejunum of pigs (Figure 3). Increasing supplemental levels of β-mannanase from 0 to 600 U/kg in the feeds did not affect expressions of occludin and claudin-1 in the jejunum of nursery pigs. However, increasing supplemental levels of β-mannanase from 0 to 600 U/kg in the feeds tended to linearly improve (*P* = 0.083) the expression of ZO-1 in the jejunum of pigs (Figure 4).

**Table 7.** Morphology and crypt cell proliferation in the duodenum and jejunum of nursery pigs fed with β-mannanase in experiment 2.

Item	β-mannanase, U^1^/kg			SEM	*P* value	
	0	400	600		Linear	Quadratic
Duodenum						
Villus height, μm	579	671	651	43	0.023	0.174
Crypt depth, μm	281	303	301	8.1	0.042	0.329
VH:CD^2^	2.06	2.20	2.15	0.12	0.313	0.379
Jejunum						
Villus height, μm	426	487	516	16	<0.001	0.944
Crypt depth, μm	175	224	246	7.8	<0.001	0.864
VH:CD	2.48	2.19	2.13	0.11	0.022	0.687
Crypt cell proliferation, %	29.3	40.7	35.5	1.99	0.014	0.011
Tight junction proteins^3^						
Occludin	0.80	0.72	0.88	0.09	0.577	0.294
Claudin-1	0.57	0.52	0.54	0.18	0.791	0.772
Zonula occludens -1	0.84	0.84	1.18	0.13	0.083	0.307

^1^One unit of β-mannanase activity is defined as the amount of enzyme required to releases 1 μmol of mannose reducing sugars equivalents per minute from locust bean gum (1.0%) in sodium phosphate buffer (200 mM), pH 6.0 at 50°C.

^2^VH:CD:villus height to crypt depth ratio.

^3^Relative band density represents each tight junction’s band ratio compared with β-action’s band density.

## Discussion

Intestinal health is comprehensive, considering the aspects of nutrient digestion and absorption, the capacity for intestinal antioxidation, immune responses, and the balance of intestinal microbiota (Jang, 2022). These factors collectively also contribute to the integrity of the intestinal barrier, a crucial aspect of nutrient utilization and a defense system against invasions by potentially harmful bacteria, toxins, or viral pathogens. The barrier function drives through complex interactions among the intestinal immune system, commensal bacteria, and the epithelial layer, effectively allowing the absorption of nutrients and transport of substances and preventing the entry of undesirable substances into the circulatory system of the body (Vancamelbeke and Vermeire, 2017). However, nursery pig generally has immature intestine insufficient to break down complex carbohydrates commonly present in feedstuffs and also susceptible to intestinal barrier dysfunction with the immune system and microbiota imbalance (Jang and Kim, 2022). In particular, β-mannans, as one of the complex carbohydrates, can contribute to an increase in digesta viscosity, which can inhibit nutrient absorption and impair the growth of pigs (Kiarie et al., 2013). β-Mannanase, an NSPase enzyme, specifically targets β-mannans and hydrolysis the β-1,4-mannosidic bonds, thereby improving the digestibility of feed ingredients and reducing digesta viscosity (Ximenes et al., 2005; Genova et al., 2023). This study shows that supplementation of β-mannanase at 361 U/kg of nursery feed could minimize the digesta viscosity in the jejunum of nursery pigs. In addition, β-mannanase supplementation showed benefits on intestinal health by reducing TNF-a, IgG, MDA, and PC, enhancing crypt cell proliferation, and increasing the expression of tight junction proteins in the jejunum of nursery pigs.

In this study, no differences in the feed efficiency might be related to possible aspects: 1) the period of supplementation was insufficient to detect the changes in feed efficiency, or 2) the baseline diet was already enough to drive improvement by the effects of β-mannanase in the short term. Future studies with longer durations, challenging conditions, or varied dietary conditions may provide further understanding of the effects of β-mannanase on feed efficiency. In this study, supplementation of β-mannanase from 0 to 600 U/kg also shows interesting patterns in BW gain of nursery pigs. Initially, as the supplemental levels were at 0 to 400 U/kg, there was a decrease of 38 g/d in ADG. However, with supplementation levels at 400 to 600 U/kg, ADG increased to 51 g/d. In other words, in the initial range, there was a decrease of 0.1 g per 1 U enzyme, but in the 400 to 600 U/kg range, there was an increase of 0.26 g per 1 U enzyme, which was opposite and more than double the initial rate. Considering the distance among the supplementation levels, this could indicate that the BW gain response to β-mannanase supplementation would fit a plateau model rather than a quadratic regression model. According to broken-line analysis, β-mannanase supplementation at over 386 U/kg could enhance the BW gain of nursery pigs. This study also shows that supplementation of β-mannanase at 361 U/kg of nursery feed could minimize the digesta viscosity in the jejunum of nursery pigs with benefits on intestinal health by reducing TNF-a, IgG, MDA, and PC, enhancing crypt cell proliferation, and increasing the expression of tight junction proteins in the small intestine of nursery pigs. These findings suggest that β-mannanase supplementation can effectively alleviate the negative effects of β-mannans on the growth and intestinal health of nursery pigs.

Digesta viscosity is critical in nutrient absorption and overall intestinal health of nursery pigs (Moita and Kim, 2022). High viscosity can impair nutrient absorption, negatively impacting growth performance and intestinal health (Chen et al., 2020; Duarte and Kim, 2021; Moita et al., 2022). The results in this study showed that supplementation of β-mannanase clearly reduced the viscosity of jejunal digesta in nursery pigs. Viscosity of jejunal digesta measured showed different values between experiments 1 and 2 possibly due to differences in NDF (12.7% in experiment 1 vs. 8.6% in experiment 2) whereas these values were within viscosity recently observed in the jejunal digesta of nursery pigs (Passos et al., 2015b; Duarte et al., 2019; Moita et al., 2022). This could be supported by the β-mannanase hydrolysis into a smaller degree of polymerization, which can be involved in the viscosity of a substance (Kauzmann and Eyring, 1940). In general, larger molecules tend to increase the viscosity compared with smaller molecules because larger molecules can have more interaction with other external compounds (Kauzmann and Eyring, 1940). Interestingly, the broken-line analysis showed that the jejunal digesta viscosity could be reduced when β-mannanase was supplemented at 360 U/kg feed, indicating that the impact on viscosity caused by β-mannans disappeared when β-mannans were degraded by β-mannanase into smaller units of mannan oligosaccharides up to a certain degree. However, the information on the degree of polymerization that increases viscosity is limited and further research is needed to explore the effect of the degree or size of the hydrolysis products on viscosity (Baker et al., 2021).

This study primarily aimed to investigate the responses to the small intestine of nursery pigs because antinutritional soluble NSP including galactomannan and β-mannans causing digesta viscosity mainly affect the small intestine of nursery pigs (Choi et al. 2023; Deng et al. 2023; Duarte et al. 2023). Thus, this study investigated the impacts of β-mannanase supplementation on responses to the small intestine where the nutrient intervention and the challenges effectively work, although β-mannanase could have potential benefits through the entire gastrointestinal tract. Hydrolysis β-mannans can effectively enhance nutrient absorption by reducing digesta viscosity and increasing the accessibility of digestive enzymes to nutrients (Chen et al., 2017; Jang et al., 2023a). However, in this study, β-mannanase supplementation did not show significant changes in nutrient digestibility of the pigs. The result could be attributed to antinutritional compounds from soybean meal as it contains not only β-mannans but also antinutritional and allergenic proteins including trypsin inhibitors, lectins, glycinin, and β-conglycinin (Hong et al., 2004; Kim et al., 2010). These antinutritional compounds in feedstuffs could exacerbate intestinal inflammation and impair nutrient digestion, potentially leading to diarrhea in nursery pigs (Li et al., 1990; Roh et al., 2015). The β-mannans are typically present in the middle lamella and primary cell wall, where β-mannans contribute to the structure and rigidity (Buckeridge, 2010; Ahl et al., 2019). The β-mannans interact with other NSP and proteins to form a complex that provides the cell strength and flexibility (Carpita and Gibeaut, 1993; Cosgrove, 2005; Scheller and Ulvskov, 2010). Previous studies have shown that minor mixed-linked mannans could combine with allergenic protein components as the primary N-linked carbohydrate structure of β-conglycinin (Koshiyama, 1966; Kimura et al., 1997). Interestingly, the levels of IgG and PC in the duodenum were numerically increased by β-mannanase supplementation, indicating that β-mannanase supplementation could potentially exacerbate immune responses and oxidative stress in the duodenum of nursery pigs when the supplementation level is insufficient. Therefore, insufficient supplementation of β-mannanase may offset the positive effects of hydrolyzing β-mannans as a part of releasing antinutritional soluble NSP on nutrient digestion, possibly due to the release of antinutritional compounds in the feeds.

Intestinal barrier function was evaluated using multiple parameters known to have roles in intestinal barrier function. The protein expression of tight junction protein was measured as the primary indicator to determine the barrier function because these proteins play critical roles in maintaining the paracellular barrier, preventing the passage of unexpected substances, such as viruses, antinutritional compounds, allergenic proteins, pathogens, etc., into the body. In addition, inflammatory cytokines (TNF-α and IL-6) and oxidative damage products (MDA and PC) as well as jejunal morphology and crypt cell proliferation were measured for this study. These parameters have been used to evaluate barrier function. In addition, the mucosal tissue from the small intestine was collected to measure intestinal localized markers of intestinal immune response and serum to evaluate systemic immune response in the body. The localized markers, including TNF-α and IgG, refer to the immune status in the small intestine, indicating direct responses to the enzyme supplementation, whereas systemic markers represent the overall immune response in the body because localized and systemic immune responses are closely linked, with immune cells and signaling molecules or proteins, to efficiently response to immunostimulants from the external environment. This connection could provide an understanding of the beneficial effects of β-mannanase on intestinal health and then connected to the immune response in the whole body. If β-mannanase changes the systemic markers without affecting the localized markers, it can be assumed that β-mannanase supplementation in feeds indirectly causes changes in the systemic immune response by affecting immune cells from other tissues. In this study, β-mannanase supplementation clearly reduced the production of both localized and systemic markers. This could indicate that β-mannanase could reduce the intestinal immune response and thus positively affect the overall immune status in the body.

Increasing supplemental levels of β-mannanase linearly reduced TNF-α, IgG, MDA, and PC both in the duodenum and the jejunum of nursery pigs. The TNF-α, a pro-inflammatory cytokine, along with IgG, an immunoglobulin, are key mediators of inflammatory response, and MDA and PC are products of oxidative damage (Duarte and Kim, 2022; Deng et al., 2023b; Jang et al., 2023b). Previous studies have shown that the decreases in TNF-α and IgG (Holanda and Kim, 2022; Deng et al., 2023a) and MDA and PC (Chen et al. 2017; Frame et al. 2020; ) concentrations in the jejunal mucosa of pigs correlate with improved growth performance and also correlate with positive changes in other indicators of intestinal health including the expressions of tight junction proteins, villi structures, and intestinal oxidative stress. The findings, which align with the changes in these indicators, suggest that β-mannanase supplementation could enhance intestinal health through reductions in TNF-α, IgG, MDA, and PC concentrations in the small intestine of the pigs. Thus, this may be partly due to mannan oligosaccharides from the hydrolysis of β-mannans which can help reduce intestinal inflammation and oxidative damage by potentially multiple mechanisms. Firstly, by promoting the growth of beneficial bacteria, mannan oligosaccharides may suppress harmful bacteria that trigger the activation of intestinal immune cells, subsequently reducing inflammatory cytokines (Yang et al., 2021). Additionally, by enhancing intestinal barrier function, mannan oligosaccharides may prevent the infiltration of pathogenic bacteria and their toxins into the circulatory, leading to a potential decrease in reducing the activation of immune cells, especially B lymphocytes for immunoglobulin production (Kim et al., 2016; Yang et al., 2021; Lu et al., 2022). According to Lu et al. (2022), the recognition of mannan oligosaccharides as pathogen-associated molecular patterns by immune cells can effectively suppress immune responses by reducing the production of cytokines and immunoglobulins. Arsenault et al. (2017) also showed that supplementation of β-mannanase to hydrolyze β-mannans positively regulates the majority of this immune-related signaling, indicating that the feed-induced immune response is suppressed by the addition of β-mannanase. Therefore, supplemental β-mannanase in swine feed hydrolyzes β-mannans, mitigating their negative impact on nutrient utilization and immune response while enhancing metabolic efficiency, thereby supporting more sustainable and antibiotic-free animal protein production (Arsenault and Kogut. 2015; Kiarie et al., 2022).

This study also shows that β-mannanase supplementation increased VH:CD and crypt cell proliferation in the jejunum of nursery pigs, indicating an enhancement in intestinal absorptive capacity. Furthermore, this study also evaluated the expression of tight junction proteins, including both cytosolic (ZO-1) and membrane-associated types (occludin and claudin-1) to evaluate the intestinal barrier integrity by β-mannanase supplementation. The ZO-1, occludin, and claudin-1 in the tight junction complex are important in maintaining barrier selectivity and permeability, essential for nutrient absorption and pathogen defense. Interestingly, the protein expression of ZO-1 tended to be increased by increasing β-mannanase supplementation. The ZO-1 is a scaffolding protein that is one of the multidomain proteins usually localized at sites of intercellular junctions and can interact with other noncytosolic tight junction proteins to regulate barrier function (Meyer et al., 2022). Based on these changes by β-mannanase supplementation, it could be expected that mannan oligosaccharides may stimulate the proliferation of enterocytes, which can enhance the structure and barrier function of the intestinal epithelium. This stimulation could be also due to the modulation of intestinal microbiota by mannan oligosaccharides for the growth of beneficial bacteria, producing short-chain fatty acids that provide energy for enterocytes and promote their proliferation (Jana et al., 2021).

In addition, the direct interaction of mannan oligosaccharides with intestinal epithelium can stimulate enterocyte proliferation. According to Mavrogeni et al. (2022), nondigestible oligosaccharides, including mannan oligosaccharides, could directly interact with intestinal epithelial and immune cells via the mitogen-activated protein kinases (MAPKs)-associated pathways involved in cellular proliferation. Furthermore, the ratio of crypt cell proliferation in the jejunum could be also partly increased by β-mannanase supplementation with the levels of MDA and TNF-α in the plasma showing minimum following the quadratic responses. Although crypt cell proliferation is important for maintaining intestinal structure and barrier function, excessive proliferation may lead to a high turnover of enterocytes, possibly resulting in immature enterocytes with increasing energy or nutrient mobilization for growth, thereby undesirably impacting growth (Williams et al., 2015). The supplementation of high levels of β-mannanase may lead to excessive production of mannan oligosaccharides that activate functional receptors on the enterocytes regarding the immune response and cell proliferation, causing overstimulation of the immune system and oxidative stress in the body (Nochta et al., 2009; Zhou et al., 2019). The overstimulation of the immune response and oxidative stress could negatively affect the health and growth of pigs, which are diverting energy from growth to immune response, causing tissue inflammation and damage, and possibly reducing the effectiveness against actual pathogens. This imbalance may also cause additional oxidative stress, leading to cellular damage. This could indicate that there is a need for careful dosage optimization to achieve beneficial effects without adverse outcomes. Therefore, this study could indicate that supplementation of β-mannanase could positively affect the intestinal structure and barrier function by activating crypt cell proliferation through direct or indirect mannan oligosaccharides interaction with the intestinal epithelium of nursery pigs.

In conclusion, supplementation of β-mannanase at 360 U/kg in the corn-soybean meal-based feeds could minimize digesta viscosity and up to 600 U/kg positively affected growth performance and intestinal health of pigs by reducing intestinal inflammation and oxidative stress and enhancing the intestinal structure and barrier function.

## Abbreviations

ADF: acid detergent fiber
ADFI: average daily feed intake
ADG: average daily gain
AID: apparent ileal digestibility
BW: body weight
cP: centipoise
DDGS: distiller’s dried grains with solubles
DM: dry matter
ELISA: enzyme-linked immunoassay
G:F: gain to feed ratio; immunoglobulin G
IgG: immunoglobulin G
MAPKs: mitogen-activated protein kinases
MDA: malondialdehyde
NDF: neutral detergent fiber
NSP: non-starch polysaccharides
PC: protein carbonyl
SID: standard ileal digestibility
TNF-α: tumor necrosis factor alpha
U: unit
VH:CD: , villus height to crypt depth
ZO-1: zonula occludens-1
